# Sociocultural factors related to the physical activity in boys and girls: PeNSE 2012

**DOI:** 10.11606/S1518-8787.2019053000516

**Published:** 2019-02-20

**Authors:** Luciano Antonacci Condessa, Otaviana Cardoso Chaves, Fernanda Marcelina Silva, Deborah Carvalho Malta, Waleska Teixeira Caiaffa

**Affiliations:** I Instituto Federal Fluminense de Educação, Ciência e Tecnologia. Itaperuna, RJ, Brasil; II Universidade Federal de Minas Gerais. Faculdade de Medicina. Observatório de Saúde Urbana de Belo Horizonte. Belo Horizonte, MG, Brasil; III Universidade Federal de Minas Gerais. Programa de Pós-Graduação em Ciências Aplicadas à Saúde do Adulto. Belo Horizonte, MG, Brasil; IV Universidade Federal de Minas Gerais. Escola de Enfermagem. Departamento de Enfermagem Materno Infantil e Saúde Pública. Belo Horizonte, MG, Brasil

**Keywords:** Adolescent, Sports, Exercise, Socioeconomic Factors, Health Surveys, Adolescente, Esportes, Exercício, Fatores Socioeconômicos, Inquéritos Epidemiológicos

## Abstract

**OBJECTIVE:**

To verify in male and female Brazilian adolescents the association of demographic, psychosocial, behavioral and sociocultural characteristics with the regular practice of physical activity.

**METHODS:**

The sample consisted of 109,104 adolescents from all Brazilian states attending the 9th year of elementary education in 2012. The response variable was the regular practice of physical activity (300+ minutes/week). The explanatory variables were grouped into four fields: demographic, psychosocial, behavioral and sociocultural. The Poisson regression was stratified by sex to evaluate the association.

**RESULTS:**

The prevalence of active adolescents was 20.2%, higher in boys (27.9%) than in girls (13.1%). It was observed a greater practice of physical activity in boys of lower age group, children of mothers with higher schooling, who consumed healthy foods such as beans, fruits, vegetables, and milk, as well as among those with family supervision. At the same time, unhealthy habits such as insomnia and alcohol consumption were also positively associated with physical activity. In girls, greater physical activity was observed among those who lived with mothers and whose mothers had higher schooling. In addition to family supervision, the practice of physical activity in girls was also positively associated with the frequency of meals with their parents. However, as in boys, insomnia and alcohol consumption were associated with an increase in the practice of physical activity.

**CONCLUSIONS:**

One-fifth of adolescents practice physical activity regularly, demonstrating the need for specific public policies to increase the percentage of active young people in the country. Maternal schooling, healthy eating habits and family supervision were associated with regular physical activity in boys and girls, evidencing the importance of the family for the acquisition of healthy habits in this age group.

## RESUMO

**OBJETIVO:**

Verificar em adolescentes brasileiros dos sexos masculino e feminino a associação de características demográficas, psicossociais, comportamentais e socioculturais com a prática regular de atividade física.

**MÉTODOS:**

A amostra foi constituída por 109.104 adolescentes de todos estados do Brasil cursando o 9° ano do ensino fundamental em 2012. A variável resposta foi a prática regular de atividade física (300+ minutos/semana). As variáveis explicativas foram agrupadas em quatro domínios: demográfico, psicossocial, comportamental e sociocultural. Para avaliar a associação, foi realizada a regressão de Poisson, estratificada por sexo.

**RESULTADOS:**

A prevalência de adolescentes ativos foi de 20,2%, maior em meninos (27,9%) do que em meninas (13,1%). Constatou-se maior prática de atividade física em meninos de menor faixa etária, filhos de mães com maior escolaridade, que consumiam alimentos saudáveis como feijão, frutas, verduras, legumes e leite, assim como entre aqueles com supervisão familiar. Ao mesmo tempo, hábitos não saudáveis como insônia e consumo de álcool também se associaram positivamente à prática de atividade física. Em meninas, foi observada maior prática de atividade física entre aquelas que viviam com as mães e cujas mães tinham maior escolaridade. Além da supervisão familiar, a prática da atividade física nas meninas também esteve associada positivamente com a frequência de realização das refeições com os pais. Entretanto, assim como nos meninos, a insônia e consumo de álcool se associaram ao aumento da prática da atividade física.

**CONCLUSÕES:**

Um quinto dos adolescentes praticam atividade física regularmente, demonstrando a necessidade de políticas públicas específicas para aumentar o percentual de jovens ativos do país. A escolaridade materna, hábitos alimentares saudáveis e supervisão familiar foram associados à prática regular de atividade física em meninos e meninas, evidenciando a importância da família para a aquisição de hábitos saudáveis nessa faixa etária.

## INTRODUCTION

The regular practice of physical activity (PA) in adolescents, defined as 60 minutes per day PA with moderate to vigorous intensity, is associated with a number of health benefits, such as increased cardiorespiratory capacity, muscular strength and bone density, as well as reduction of obesity, levels of lipoprotein and depression^1^.

Assessing the percentage of active young people in several countries, it is verified that only 19.5% of adolescents practice PA regularly^2^. In Brazil, the *Pesquisa Nacional de Saúde Escolar* (National School Health Survey – PeNSE), conducted with 102,072 adolescents, found that 20.3% of them are active^3^. These results are worrying, since the habits acquired by these young people tend to remain in adult life^3^ ^–^ ^5^.

Some studies, especially those using an ecological model, show that increasing of PA practice is a complex process determined by a relation of individual factors (biological and psychological), interpersonal (family social support and cultural norms) and environmental (violence and climate), besides aspects related to national policy (urban planning and parks construction) and global (economic development)^6^. These factors have been grouped according to their characteristics in the demographic fields (sex and skin color), psychosocial (self-efficacy and stress), behavioral (smoking and eating habits), sociocultural (family support for PA) and environmental (proximity to parks)^6^.

Certain demographic characteristics of the adolescent, such as being male, with a lower age group and a mother with a higher educational level, are already well established in the literature and associated with regular practice of PA^5^ ^,^ ^7^ ^,^ ^8^. A study demonstrates that family composition interferes in the level of PA of adolescents, but differently in boys and girls^9^. The importance of health-related behaviors in the practice of PA is also highlighted. Fernandes et al.^10^ revealed that the increase in PA is associated with healthy eating habits while Bedendo and Noto^11^ reported an increase in alcohol consumption associated with this practice.

Since these associations may vary among boys and girls, and there are few researches investigating associations of these and other factors with PA in representative samples from all over Brazil, this study aimed to verify the association of demographic, psychosocial, behavioral and sociocultural characteristics with the regular practice of PA in Brazilian male and female adolescents. Understanding these relationships may contribute to the development of intervention programs that aims increasing the prevalence of active youth.

## METHODS

This is a cross-sectional study, with information from the second edition of PeNSE, conducted with 109,104 adolescents in 2012^4^. PeNSE 2012 is a partnership between the Ministry of Health and the Brazilian Institute of Geography and Statistics, with the support of the Ministry of Education, and aimed to investigate risk factors and health protection of adolescents. The students from ninth year of elementary education, mostly aged between 13 and 15 years, from public or private schools located in urban or rural areas of a set of Brazilian municipalities were the target population of the research. The selection of the sample was made based on the register of schools ranked by the 2010 School Census, conducted by the National Institute of Studies and Educational Research Anísio Teixeira, Ministry of Education, excluding schools with fewer than 15 students in that school year^4^.

Sample design was structured to estimate proportions or prevalence in the following geographic fields: 26 Brazilian capitals and the Federal District, all these capitals, five regions of the country (North, Northeast, Southeast, South and Midwest) and Brazil as a whole. Further details on sample design are available at PeNSE 2012^4^.

PeNSE 2012 data collection was performed using a smartphone containing a structured and self-administered questionnaire divided into modules by subject: sociodemographic characteristics; job; food; body image; PA; consumption of tobacco, alcohol and other drugs; safety nets; hygiene habits; mental health; oral health; asthma; sexual behavior; violence; accidents; and use of health services^4^.

### Description of Variables

In this study, the response variable was the regular practice of PA, measured by the globally estimated physical activity indicator, using the question: “How many days did you have PA for at least 60 minutes (one hour) on the last seven days?”. Adolescents who did at least 60 minutes of PA in five days or more were considered active. This variable was categorized as insufficiently active (< 300 min/week) and active (≥ 300 min/week)^4^ ^,^ ^8^ ^,^ ^9^.

Explanatory variables included in the study were grouped, by affinity, into four fields^6^. In the demographic field, the following variables were evaluated: age group (≤ 13; 14; 15; ≥ 16 years), skin color (white; black; yellow; brown; indigenous)^8^, kind of school (private; public)^8^, current adolescent work (yes; no)^8^, mother's schooling (without schooling; incomplete or complete elementary education; incomplete or complete secondary education; incomplete or complete higher education)^12^, living with parents (live with the mother and the father; only with the father; only with the mother; do not live with father or mother)^9^.

In the psychosocial field, the following variables were evaluated: feeling of loneliness^4^ (never, rarely, sometimes; often, always), report of insomnia (never, rarely, sometimes; often, always)^4^ and the existence of close friends (no, one friend or more)^4^.

The variables evaluated in the behavioral field were: regular consumption of healthy foods such as beans, fruits, vegetables and milk (five times or more in the week)^4^, regular use of tobacco and alcohol in the last 30 days (never; none day; one or more days)^4^ and if they ever tried drugs (no; yes)^4^.

In the sociocultural field, were evaluated: frequency of meals in the presence of the mother, father or guardian (four or fewer meals per week or five meals or more per week)^4^; family supervision, defined as the knowledge of guardians about what the adolescent does in free time (never, rarely, sometimes or often/always)^4^; report of domestic physical aggression with the adolescent as the victim (not once in the last 30 days or once or more in the last 30 days)^4^; reaction of parents or guardians if the adolescent arrived drunk, according to the adolescent (they would care a little, they would not care, do not know, they would care a lot)^4^; absences at school without parental or guardian permission (none day in the last 30 days or one to ten days in the last 30 days)^4^.

Additionally, the socioeconomic status was analyzed through a score of goods and services, categorized in tertiles. In this approach, we considered the goods and services with a lower prevalence than 70% (landline, computer, internet access, car, motorcycle, shower, maid). Weights were assigned to the presence of each of them, corresponding to the complement of the relative frequency in the sample studied. Thus, the items that were less frequent received a greater weight^13^.

### Data Analysis

First, a descriptive analysis was performed, presenting the proportions of the variables according to the adolescents’ PA, stratified by sex. Bivariate Poisson regression was performed to verify the factors associated with PA, with estimates of the prevalence ratio (PR) and their respective trust intervals (95%CI). The variables that presented p < 0.20 in the bivariate were included in the multivariate model; the only variable included even without this criterion was the regular use of tobacco in both sexes, due to its epidemiological relevance. In the final model the variables with p ≤ 0.05 remained.

The suitability of the models was verified by goodness-of-fit. Analyzes were done in Stata software version 12.0 (Stata Corporation, College Station, USA), considering the complexity of the sample design. In addition, the goods and services score was used to assess if the sample loss of the mother's educational variable was differential or not.

### Ethical Aspects

PeNSE 2012 was approved by the National Commission for Research Ethics under registry number 16805. The conduct of the research is in accordance with the Declaration of Helsinki, with the voluntary participation of the adolescents and all information, both student and school, confidential and unidentified.

## RESULTS

Table 1 presents the descriptive analysis of the explanatory variables used in this study. PeNSE 2012 evaluated 109,104 adolescents, most of them female (52.2%), aged 14 years old (45.5%) and public school student (82.8%). Regular PA practice was observed in 20.2% (95%CI 19.6–20.7) of adolescents, being more prevalent in boys (27.9%, 95%CI 27.0–28.8) than girls (13.1%, 95%CI 12.6–13.6), with p ≤ 0.05.

**Table 1:** Demographic, psychosocial, behavioral and sociocultural characteristics of Brazilian adolescents.

Variable		Total		Male		Female	
		%	95%CI	%	95%CI	%	95%CI
Age group (years old)							
	≤ 13	22.9	17.0–28.8	19.8	13.8–25.8	25.8	19.8–31.8
	14	45.5	42.4–48.7	43.9	39.9–47.9	47.1	44.4–49.7
	15	18.4	14.4–22.3	20.5	16.7–24.3	16.4	12.2–20.6
	≥ 16	13.2	8.4–18.0	15.8	9.9–21.8	10.8	6.7–14.8
Skin color							
	White	36.8	31.6–42.0	38.7	33.3–44.1	35.0	30.1–40.0
	Black	13.4	11.1–15.6	15.5	13.0–18.0	11.4	9.2–13.5
	Yelow	4.1	3.4–4.8	3.8	3.4–4.1	4.4	3.3–5.6
	Brown	42.2	39.1–45.4	38.5	35.3–41.6	45.7	42.6–48.8
	Indigenous	3.5	2.8–4.3	3.6	2.7–4.5	3.5	2.8–4.2
Kind of school							
	Public	82.8	78.2–87.4	82.3	76.9–87.7	83.3	79.4–87.3
	Private	17.2	12.6–21.8	17.7	12.3–23.1	16.7	12.7–20.6
Work currently							
	Yes	13.1	12.2–14.1	17.4	16.1–18.8	9.2	8.4–10.0
	No	86.9	85.9–87.8	82.6	81.2–83.9	90.8	90.0–91.6
Mother's schooling							
	Without schooling	10.1	8.3–11.9	9.7	8.4–10.9	10.4	8.1–12.8
	Elementary education (incomplete/complete)	41.8	39.9–43.8	40.3	37.9–42.7	43.2	41.1–45.3
	Secondary education (incomplete/complete)	31.8	28.3–35.4	32.2	28.5–35.9	31.5	27.8–35.2
	Higher education (incomplete/complete)	16.3	14.7–17.8	17.8	16.4–19.2	14.8	13.3–16.4
Living with parents							
	Do not live with parents	5.4	5.0–5.8	4.6	4.1–5.1	6.1	5.7–6.5
	Only father	4.0	3.8–4.1	4.8	4.6–5.0	3.2	3.1–3.4
	Only Mother	28.4	26.9–30.0	26.5	25.2–27.8	30.2	28.4–32.0
	Both	62.2	60.2–64.2	64.0	62.3–65.8	60.5	58.3–62.7
Feeling of loneliness							
	Yes	16.5	16.1–16.8	10.7	10.4–11.0	21.7	21.2–22.2
	No	83.6	83.2–83.9	89.3	89.0–89.6	78.3	77.8–78.8
Insomnia							
	Yes	9.7	9.3–10.1	6.3	6.2–6.5	12.8	12.1–13.5
	No	90.3	89.9–90.7	93.7	93.5–93.8	87.2	86.5–87.9
Close friends							
	No	3.5	3.3–3.7	4.6	4.2–5.0	2.5	2.4–2.6
	1 friend or more	96.5	96.3–96.7	95.4	95.0–95.8	97.5	97.4–97.6
Regular consume of beans (≥ 5x/week)							
	No	30.1	27.4–32.8	25.3	22.6–28.0	34.4	31.3–37.5
	Yes	69.9	67.2–72.6	74.7	72.0–77.4	65.6	62.5–68.7
Regular consume of fruits (≥ 5x/week)							
	No	69.8	68.9–70.7	70.4	69.8–71.1	69.2	68.1–70.3
	Yes	30.2	29.3–31.1	29.6	28.9–30.2	30.8	29.7–31.9
Regular consume of vegetables (≥ 5x/week)							
	No	65.2	63.3–67.2	66.2	64.4–67.9	64.4	62.2–66.5
	Yes	34.8	32.8–36.7	33.8	32.1–35.6	35.6	33.5–37.8
Regular consume of milk (≥ 5x/week)							
	No	48.5	45.2–51.7	45.3	41.7–49.0	51.3	48.4–54.3
	Yes	51.6	48.3–54.8	54.7	51.0–58.3	48.7	45.7–51.6
Regular consume of tobacco							
	Yes	5.1	3.9–6.2	5.1	4.4–5.9	5.0	3.5–6.6
	No	94.9	93.8–96.1	94.9	94.1–95.6	95.0	93.4–96.5
Regular consume of alcohol							
	Yes	26.1	24.5–27.7	25.2	23.0–27.5	26.9	25.7–28.0
	No	73.9	72.3–75.5	74.8	72.5–77.0	73.1	72.0–74.3
Drugs trying							
	Yes	7.1	5.0–9.1	7.9	5.8–9.9	6.3	4.3–8.3
	No	92.9	90.9–95.0	92.1	90.1–94.2	93.7	91.7–95.7
Frequency of meals with parents/guardian							
	4 fewer meals per week	33.6	31.6–35.6	30.9	29.4–32.5	36.0	33.5–38.6
	5 meals or more per week	66.4	64.4–68.4	69.1	67.5–70.6	64.0	61.4–66.5
Family supervision							
	No	41.6	41.0–42.1	45.5	44.7–46.2	37.9	37.3–38.6
	Yes	58.5	57.9–59.0	54.5	53.8–55.3	62.1	61.4–62.7
Victim of domestic physical aggression							
	Yes	10.6	10.1–11.1	9.6	8.2–11.0	11.5	107–12.3
	No	89.4	88.9–89.9	90.4	89.0–91.8	88.5	87.7–89.3
Reaction of parents or guardians if the adolescent arrived drunk							
	They would care a little	10.3	10.1–10.4	11.3	11.0–11.6	9.4	9.1–9.7
	They would care so much	89.7	89.6–89.9	88.7	88.4–89.0	90.6	90.3–90.9
Absences at school without parental or guardian permission							
	Yes	25.8	24.6–27.0	28.0	26.5–29.4	23.8	22.6–25.0
	No	74.2	73.0–75.4	72.0	70.6–73.5	76.2	75.0–77.4

The mother's schooling was absent for 18.6% of adolescents. However, this loss was non-differential (difference of a maximum of 4%) when comparing the groups of students who knew and did not know the mothers’ educational level, considering gender, age group, type of school, score of goods and services (in tertiles) and PA.

Bivariate analysis is described in Table 2 and indicates the factors associated with regular practice of PA, stratified by sex. In the multivariate analysis described in Table 3, a higher prevalence of PA was observed in 14-year-old boys, with brown skin color, children of mothers with some level of schooling, who frequently consumed beans, fruits, vegetables and milk, who did not smoke regularly and had family supervision. On the other hand, there was a lower prevalence of PA in male adolescents who did not work, did not present insomnia, did not consume alcohol regularly and did not try drugs (Table 3).

**Table 2:** Factors associated with the regular practice of physical activity of Brazilian adolescents, according to the demographic, psychosocial, behavioral and sociocultural characteristics. Bivariate analysis.

Variable		Boys			Girls		
		PR	95%CI	p	PR	95%CI	p
Age group							
	≥ 16	1			1		
	15	1.08	1.01–1.15	**0.019**	0.93	0.83–1.03	0.171
	14	1.11	1.07–1.16	**0.000**	0.97	0.86–1.08	0.537
	≤ 13	1.06	0.98–1.14	0.140	0.99	0.87–1.13	0.870
Skin color							
	White	1			1		
	Black	0.98	0.90–1.06	0.649	0.92	0.86–0.99	**0.029**
	Yelow	0.97	0.80–1.19	0.797	1.11	0.99–1.25	0.072
	Brown	1.04	0.97–1.10	0.251	0.94	0.86–1.03	0.210
	Indigenous	1.08	0.99–1.18	0.102	1.18	1.04–1.34	**0.011**
Kind of school							
	Public	1			1		
	Private	1.06	1.00–1.11	0.037	1.18	1.09–1.27	**< 0.001**
Work currently							
	Yes	1			1		
	No	0.85	0.80–0.89	**< 0.001**	0.74	0.70–0.77	**< 0.001**
Mother's schooling							
	Without schooling	1			1		
	Elementary education (incomplete/complete)	1.12	1.08–1.16	**< 0.001**	1.02	0.92–1.13	0.742
	Secondary education (incomplete/complete)	1.19	1.14–1.24	**< 0.001**	1.13	1.06–1.21	**< 0.001**
	Higher education (incomplete/complete)	1.43	1.34–1.52	**< 0.001**	1.48	1.39–1.57	**< 0.001**
Living with parents							
	Do not live with parents mother	1			1		
	Only father	0.95	0.86–1.06	0.373	1.09	0.90–1.32	0.375
	Only Mother	0.93	0.83–1.05	0.231	1.09	0.96–1.24	0.167
	Both	0.96	0.86–1.06	0.397	1.01	0.92–1.12	0.802
Feeling of loneliness							
	Yes	1			1		
	No	1.01	0.95–1.08	0.671	0.95	0.90–1.01	0.116
Insomnia							
	Yes	1			1		
	No	0.87	0.83–0.91	**< 0.001**	0.84	0.73–0.97	**0.020**
Close friends							
No		1			1		
1 friend or more		1.08	1.03–1.13	**0.002**	0.97	0.82–1.15	0.726
Regular consume of beans (≥ 5x/week)							
	No	1			1		
	Yes	1.25	1.20–1.31	**< 0.001**	1.14	1.06–1.23	**< 0.001**
Regular consume of fruits (≥ 5x/week)							
	No	1			1		
	Yes	1.58	1.52–1.65	**< 0.001**	1.92	1.79–2.06	**< 0.001**
Regular consume of vegetables (≥ 5x/week)							
	No	1			1		
	Yes	1.57	1.52–1.61	**< 0.001**	1.72	1.60–1.85	**< 0.001**
Regular consume of milk (≥ 5x/week)							
	No	1			1		
	Yes	1.32	1.27–1.38	**< 0.001**	1.43	1.36–1.50	**< 0.001**
Regular consume of tobacco							
	Yes	1			1		
	No	0.98	0.92–1.05	0.568	0.87	0.70–1.08	0.212
Regular consume of alcohol							
	Yes	1			1		
	No	0.88	0.85–0.91	**< 0.001**	0.90	0.86–0.95	**< 0.001**
Drugs Trying							
	Yes	1			1		
	No	0.90	0.84–0.96	**0.001**	0.80	0.71–0.89	**< 0.001**
Frequency of meals with parents/guardian							
	4 fewer meals per week	1			1		
	5 meals or more per week	1.08	1.01–1.15	**0.015**	1.12	1.08–1.15	**< 0.001**
Family supervision							
	No	1			1		
	Yes	1.24	1.21–1.28	**< 0.001**	1.26	1.16–1.38	**< 0.001**
Victim of domestic physical aggression							
	Yes	1			1		
	No	0.96	0.91–1.01	0.103	0.85	0.78–0.91	**< 0.001**
Reaction of parents or guardians if the adolescent arrived drunk							
	They would care a little	1			1		
	They would care so much	0.97	0.89–1.04	0.374	0.84	0.78–0.90	**< 0.001**
Absences at school without parental or guardian permission							
	Yes	1			1		
	No	1.02	0.98–1.07	0.297	1.05	0.99–1.11	0.086

Numbers with p-valor ≤ 0.05 in bold.

**Table 3:** Factors associated with the regular practice of physical activity, stratified by sex, according to the demographic, psychosocial, behavioral and sociocultural characteristics.

Variable		Boys			Girls		
		PR	95%CI	p	PR	95%CI	p
Age group							
	≥ 16	1					
	15	1.02	0.96–1.09	0.576			
	14	1.06	1.03–1.10	0.001			
	≤ 13	0.99	0.94–1.06	0.836			
Skin color							
	White	1			1		
	Black	0.99	0.93–1.07	0.886	1.03	0.95–1.12	0.456
	Yelow	0.98	0.81–1.18	0.801	1.19	1.06–1.35	0.005
	Brown	1.07	1.02–1.13	0.008	1.04	0.97–1.12	0.268
	Indigenous	1.10	0.98–1.24	0.100	1.26	1.05–1.51	0.011
Work currently							
	Yes	1			1		
	No	0.87	0.83–0.92	< 0.001	0.76	0.73–0.79	< 0.001
Mother's schooling							
	Without schooling	1			1		
	Elementary education (incomplete/complete)	1.05	1.00–1.10	0.041	0.96	0.86–1.07	0.436
	Secondary education (incomplete/complete)	1.09	1.04–1.14	< 0.001	1.03	0.97–1.09	0.404
	Higher education (incomplete/complete)	1.26	1.20–1.31	< 0.001	1.27	1.19–1.35	< 0.001
Living with parents							
	Do not live with father or mother				1		
	Only father				1.18	0.99–1.41	0.058
	Only Mother				1.15	1.01–1.32	0.035
	Both				1.07	0.96–1.19	0.235
Insomnia							
	Yes	1			1		
	No	0.91	0.84–1.00	0.044	0.89	0.80–1.00	0.046
Regular consume of beans (≥ 5x/week)							
	No	1					
	Yes	1.14	1.11–1.17	< 0.001			
Regular consume of fruits (≥ 5x/week)							
	No	1			1		
	Yes	1.38	1.31–1.44	< 0.001	1.63	1.52–1.75	< 0.001
Regular consume of vegetables (≥ 5x/week)							
	No	1			1		
	Yes	1.35	1.30–1.40	< 0.001	1.39	1.31–1.47	< 0.001
Regular consume of milk (≥ 5x/week)							
	No	1			1		
	Yes	1.19	1.16–1.22	< 0.001	1.25	1.20–1.30	< 0.001
Regular consume of tobacco							
	Yes	1					
	No	1.11	1.00–1.23	0.050			
Regular consume of alcohol							
	Yes	1			1		
	No	0.85	0.80–0.89	< 0.001	0.92	0.86–0.98	0.012
Drugs trying							
	Yes	1			1		
	No	0.91	0.83–0.99	0.035	0.82	0.72–0.93	0.002
Frequency of meals with parents/guardian							
	4 fewer meals per week				1		
	5 meals or more per week				1.06	1.01–1.10	0.017
Family supervision							
	No	1			1		
	Yes	1.17	1.12–1.22	< 0.001	1.22	1.10–1.34	< 0.001
Victim of domestic physical aggression							
	Yes				1		
	No				0.85	0.80–0.91	< 0.001
Reaction of parents or guardians if the adolescent arrived drunk							
	They would care a little				1		
	They would care so much				0.87	0.79–0.97	0.010

Likewise, a higher prevalence of PA was observed in girls with yellow and indigenous skin color, daughters of mothers with higher education, daughters who lived with the mother, frequently consumed fruits, vegetables and milk, had five or more meals per week with parents or guardians and had family supervision. However, a lower prevalence of PA was found in girls who did not work, did not present insomnia, did not consume alcohol regularly, did not try drugs, did not suffer aggression from a relative and whose parents would care a lot if they got drunk at home. It should be noted that the models for boys and girls presented adequate adjustment.

## DISCUSSION

This is the first Brazilian study, with national representativeness, that found, in adolescents of both sexes, a greater regular practice of PA in teenagers of mothers with higher schooling and under family supervision, showing a possible influence of the family structure in the practice of PA of these youngsters. In addition, PA was positively associated with healthy eating habits such as regular consumption of fruits, vegetables and milk. At the same time, alcohol consumption was prevalent in active adolescents, showing the importance of discussing public policies to prevent this undesirable behavior.

In the present study, it was verified that only 20.2% of Brazilian adolescents practice PA regularly. Comparing the prevalence of active adolescents in Brazil with those from other countries, it is observed that Brazilians are more active than Ghanaians (16.0%) and less active than the Argentines and Uruguayans (23.3% and 27.0%, respectively)^14^. This low proportion of assets, both in Brazil and in other countries, can be explained partly by the rapid urbanization and the scarcity of public policies to encourage PA, showing the importance of promotion measures to prevent the development of chronic non-communicable diseases in adulthood^1^ ^,^ ^3^ ^,^ ^15^.

Age is known as a factor that influences the practice of PA among adolescents, with a tendency of younger age groups to be more active than older ones^5^. A study by Silva et al.^16^ shows that 16 year old adolescents are more absent from Physical Education classes than 15 year old ones, contributing to the greater sedentary lifestyle of the first group. This may be one of the plausible explanations for the greater percentage of assets among 14 year old boys than 16 year old ones. In a stratified analysis (data not shown), we found that 14 year old boys had more physical education classes than 16 year old ones (PR = 1.10; 95%CI 1.02–1.18). The same did not occur among the girls (PR = 1.10, 95%CI 0.90–1.33).

In Brazilian adolescents, the association between PA and skin color has presented inconsistent results^7^ ^,^ ^17^. While Farias Junior et al.^7^ found no association between the two variables in schoolchildren living in João Pessoa, our study, analyzing adolescents from all over the country, found a higher level of PA in brown skinned boys and in yellow and indigenous skinned adolescents compared with white skinned adolescents. Rezende et al.^8^ had already found association of yellow and brown skin color with active commuting, but their analysis was not stratified by sex. Another study showed that the indigenous population presents a higher level of general PA^18^. In a stratified analysis (data not shown) using the last tertile of goods and services, a lower socioeconomic level was observed in adolescents with brown (23.0%), yellow (30.9%) and indigenous (26.6%) skin than white ones (37.5%), which is a possible justification for the higher PA due to active commuting, in view of the lower access to goods and services.

In addition, commuting is probably also responsible for the association between PA and work. Recently, Rezende et al.^8^ found that working adolescents had higher levels of active commuting than those who did not work, probably because the first group belongs to a lower socioeconomic class, who moves to school more often by bicycle or on foot.

The relationship found between higher maternal schooling and regular practice of PA by adolescents can be elucidated by the higher financial level of higher educated families, which would contribute to higher leisure PA due to access to walking and jogging tracks, cycle paths, squares and sport modalities practiced in private places such as clubs^5^ ^,^ ^7^. Thus, government programs that encourage the increasing of schooling are required, because they may indirectly increase the prevalence of active adolescents.

The presence of the mother or father at home (at the p-value limit of 0.058) was associated with regular practice of PA by girls. The influence of family composition on PA had already been demonstrated in another study^9^. In a stratified analysis (data not shown), we found a greater leisure PA in girls who lived with mother or father (PR = 1.08; 95%CI 1.03–1.14) compared to those who do not have parents at home (PR = 1.07; 95%CI 1.02–1.12). This association was not observed among boys, that may be related to the higher determination of this group for the practice of PA in relation to girls^19^, reducing the influence of parents in male adolescents.

The association of insomnia with PA was an unusual result found in our study. Insomnia in adolescents has been attributed to an interaction of intrinsic factors (physical, psychological and social changes) and extrinsic (beginning of schooling) at puberty ^20^. In recent literature review, Lang et al.^20^ found that adolescents who do more PA have better sleep quality and therefore less insomnia. Since the PeNSE questionnaire did not evaluate the physical changes and not all the psychological and social variables, this association may have occurred due to the presence of residual confounding factors that remained even after adjustment. It is also emphasized that insomnia was measured by means of a self-reported questionnaire containing only one question about the difficulty of sleeping at night, an important limitation. Another possibility would be the presence of reverse causality, considering that adolescents with insomnia can adopt this practice as a way to reduce it^20^ ^–^ ^22^. However, further investigations are required, since this association was maintained in both sexes.

The aggregation of behaviors considered healthy, such as the high level of PA and healthy eating, reported in the present study, has been described in studies evaluating young people. One study found that high levels of PA were associated with regular consumption of vegetables and fruits^10^. In addition, inadequate consumption of fruits, vegetables and greens was associated with low levels of PA in adolescents^23^. Unhealthy habits related to diet and PA and habits considered healthy seem to be concomitant. Then, public policies that invest in increasing of the practice of PA can contribute to the development of healthy eating habits in adolescents.

Regarding the risk behaviors, PA is a protective factor against smoking in male adolescents according this study. Although there are doubts about the relationship between smoking and PA^6^, our results agree with those of Ali et al.^24^, who also found lower tobacco consumption among active adolescents, probably because smoking reduces the aerobic capacity required for the performance of most sports activities^25^.

Unlike cigarettes, in our study we found a positive association of PA with alcohol consumption and drug trying in both sexes. These results corroborate the study made by Bedendo and Noto^11^, who also verified greater consumption of alcohol among adolescents attending fitness centers and soccer players in all Brazilian capitals. In another study it was observed that adolescents who participated in sports activities were more likely to abuse alcohol in adulthood^25^. One of the possible explanations is that alcohol consumption can be encouraged among friends who practice PA as a way to strengthen group bonds by facilitating socialization^26^ ^,^ ^27^. Therefore, it is suggested that public policies aimed at increasing the practice of PA in adolescents should adopt preventive measures to reduce alcohol consumption.

With regard to illicit drugs trying, our result differs from that found by Silva et al.^16^, who did not observe an association of this factor with PA in 6,000 adolescents from the state of Santa Catarina. This divergence can be attributed to the local differences, since the sample of this study is representative of all Brazil. Unlike Silva et al.^16^ and similarly to our results, Horta et al.^12^, when analyzing adolescents from all Brazilian states, found a positive association of illicit substance trying with PA and number of friends. However, it is a matter of trying and not regular use of drugs, this association should be analyzed with caution and more studies are necessary to prove the relationship between these factors.

When analyzing the sociocultural field, more specifically the family context, we found that girls who reported more meals with their parents had a higher chance of being active. Some studies shown that eating more than five meals a week with the family contributes to improving the health and well-being of adolescents^28^. However, it is noteworthy that such association was found only in girls. Possible explanations fall on sex differences related to the greater sensitivity and susceptibility of girls in family relationships^19^ ^,^ ^28^. Recently, Banna et al.^29^ reported that parental guidance influences the choice of healthy foods among adolescents, demonstrating that parent-child dialogue during meals is an incentive for healthy lifestyle habits, possibly including PA.

Another important part of the possible family influence in the practice of PA is represented by the family structure. Berge et al.^30^ demonstrated that boys living in well-structured families have a higher practice of PA, so in our study, boys and girls with family supervision were more likely to be active than the others. This association, coupled with previous ones, shows that government programs that encourage family ties and positive social relationships in this nucleus can contribute to increased practice of PA as well as other healthy habits.

Family aggression and parents who would not care about their children's abusive alcohol consumption were associated with increased regular practice of PA among girls, associations not expected by the authors of the article, since positive family relationships, as described in the previous paragraphs, tend to increase PA practice in adolescents. The explanation may be in the possible residual confounding factors present even after adjusting for several variables.

Among the limitations of the research, it is emphasized that the collection of information through a questionnaire can introduce information bias for most of the variables, including the outcome. Moreover, due to the impossibility of establishing a causal relationship, longitudinal studies for causal inferences are strongly recommended. To the detriment of the limitations, the greatest advantage of this study is its power and sample amplitude, being representative of Brazil.

It was concluded that PA was associated with several demographic, psychosocial, behavioral and sociocultural factors. The positive association of maternal schooling, family supervision and healthy eating habits with the regular practice of PA in boys and girls is particularly noteworthy, which may lead to the elaboration of public policies to promote PA. At the same time, regular alcohol consumption was also associated with PA, highlighting the need to adopt preventive measures to control this behavior.
